# Suitability of Electrodialysis with Monovalent Selective Anion-Exchange Membranes for Fractionation of Aqueous Mixture Containing Reactive Dye and Mineral Salt

**DOI:** 10.3390/membranes15030085

**Published:** 2025-03-07

**Authors:** Katarzyna Majewska-Nowak, Arif Eftekhar Ahmed, Martyna Grzegorzek, Karolina Baraniec

**Affiliations:** 1 Faculty of Environmental Engineering, Wrocław University of Science and Technology, Wybrzeże Wyspiańskiego 27, 50-370 Wrocław, Poland; arif.eftekhar.ahmed@pwr.edu.pl (A.E.A.); martyna.grzegorzek@pwr.edu.pl (M.G.); 2 NIRAS, Strzegomska 138, 54-429 Wrocław, Poland; baranieckaro@gmail.com

**Keywords:** reactive dye, fractionation, electromembrane process, monovalent selective anion-exchange membrane

## Abstract

To fulfil the goals of the circular economy, the treatment of textile wastewater should be focused on the recovery of valuable components. Monovalent anion-selective electrodialysis (MASED) was applied for the separation of reactive dyes from mineral salts. Standard cation-exchange membranes (CM membranes) and monovalent selective anion-exchange membranes (MVA membranes) were used in the electrodialysis (ED) stack. The separation efficiency was evaluated for model solutions of various reactive dyes (varying in molecular weight and chemical reactivity) containing NaCl. In the course of MASED, the mineral salt was successfully removed from the dye solutions with an efficacy of 97.4–99.4%, irrespectively of the composition of the treated solution. The transport of dye molecules through the ion-exchange membranes (IEMs) from diluate to concentrate compartments was irrelevant. Nonetheless, a significant adsorption of dye particles on the membranes was observed. Around 11–40% of the initial dye mass was deposited in the ED stack. Dye adsorption intensity was significantly affected by dye reactivity. This study showed the potential of the MASED process for the separation of the reactive dye from the mineral salt on condition that antifouling membrane properties are improved. The obtained streams (the concentrate rich in mineral salt and the diluate containing the reactive dye) can be reused in the dye-house textile operations; however, some loss of dye mass should be included.

## 1. Introduction

The use of reactive dyes in textile manufacturing has increased due to their desirable properties. Reactive dyes are water-soluble, anionic in nature, and they can form strong covalent bonds with cellulose fibres. These dyes are widely used due to their excellent wash fastness, vivid colours, and ease of application. They are particularly useful in the textile industry for their vibrant shades and ability to resist fading over time. The dyeing process involves applying the dye to the fabric in an alkaline solution, which activates the dye’s reactive groups and facilitates the formation of a strong covalent bond between the dye and the fibre [1,2]. The presence of electrolytes in a dye bath reduces the extent of the electrostatic repulsion between the ionized dyes and fibre, thereby increasing the substantivity of dyes [3]. A dye bath used for dyeing cotton fibre usually contains 300–800 mg/L of reactive dyes, as well as 4–10 (up to even 80) g/L of NaCl. However, around 20–50% of the dyes applied are not fixed on the textile substrate [4,5]. Consequently, the unfixed dyes are present in textile wastewater together with other auxiliary chemicals (salts, acids, hydroxides, dispersing and complexing agents, etc.).

The discharge of dye-containing wastewater into the environment has raised concerns due to the harmful effects of these chemicals on both human health and ecosystems. Reactive dyes can inhibit the photosynthesis process in water bodies by blocking sunlight penetration. The deposition of these dyes in soil can lead to the accumulation of toxic substances in crops. Direct contact with reactive dyes can cause skin irritation, rashes, and allergic reactions. Some reactive dyes have been found to possess mutagenic and carcinogenic properties [6,7]. Therefore, the presence of reactive dyes in the environment is a serious threat, and the proper management of dye-containing effluents should be implemented unconditionally. Moreover, this management should be performed under the rules of the closed-loop economy, focusing on the recovery of water together with organic dyes and minerals reuse. It seems that most of the common wastewater treatment methods (biodegradation, flocculation-coagulation, chemical precipitation, adsorption, and advance oxidation) are rather useless in meeting the rules of the circular economy. However, membrane technology emerges as especially suitable for the valorisation of dye-house effluents, enabling the reuse of water and chemicals in technological processes.

Among various membrane techniques, pressure membrane processes such as reverse osmosis (RO) and nanofiltration (NF) are the most common methods of textile wastewater treatment. These methods have been applied for more than 2 decades, although they have many drawbacks (high energy demand, membrane fouling, and high divalent salt rejection), which make RO/NF unsuitable for the efficient fractionation of dye-house wastewater [7,8,9].

Electromembrane processes are recognized as a membrane technology of great application potential. Treatment methods involving electrodialysis (ED) are useful not only for the simple desalination of brackish water but also for the fractionation of various salt mixtures and the valorisation of industrial wastewater. Furthermore, this technology is continuously developing, and novel specific membranes (monovalent selective ion-exchange membranes and bipolar membranes) are produced as the basis of new ED processes such as selective electrodialysis (SED), bipolar membrane electrodialysis (BMED), electrodeionization (EDI), electrodialysis metathesis (EDM), and electro-electrodialysis bipolar membrane (EEDBM) [10,11]. Generally, all these processes use ion-exchange membranes (IEMs), which enable charged solutes to pass through the membranes, while uncharged components are retained. Thus, electromembrane processes are widely used for the recovery of mineral and organic acids, whey desalination, the deacidification of fruit juice, the desalination of protein products and amino acids, and the production of lactic acid [10,11,12]. More recently, a novel SED process has been used to recover nutrients from municipal wastewater or divalent metal ions from metallurgical process water [12]. Due to the application of the monovalent ion-selective membranes, the SED process is very efficient in the fractionation of monovalent ions from divalent ions of the same sign.

The possibility of the application of electromembrane processes (ED, SED, and BMED) in dye-house effluent valorisation has been noticed by some researchers, but not too many. Majewska-Nowak [13] investigated conventional electrodialysis (ED) to separate direct dyes from mineral salts. The desalination efficiency for all dye solutions was very high (98%); however, it was observed that some dyes penetrated into the membranes, and their concentration in the diluate diminished with process time. At the end of the process, the decrease in dye concentration in the diluate cells varied from about 50% (for direct dyes of a molecular weight in the range of 800–1000 Da) to even 80–90% (for dyes of a molecular weight in the range of 300–600 Da). This phenomenon was clarified by the deposition of dye molecules in the membrane matrix. To overcome this strong dye adsorption, the SED process with conventional cation-exchange membranes (CM membranes) and monovalent anion-selective membranes (MVA membranes) was investigated. It was assumed that the MVA membrane possessing an additional polymer layer with a negative charge would be an effective barrier for dye macro-anion migration through an anion-exchange membrane into concentrate cells. Furthermore, it was also expected that due to the electrostatic repulsion between an anionic dye particle and the negative surface charge of the MVA membrane, the dye adsorption and organic membrane fouling would be markedly reduced. The SED process allowed the satisfactory desalination of anionic dye solution, irrespectively of the direct dye type, although the desalination effect was slightly lower in comparison to the desalination efficiency for the standard ED (96% and 98% for SED and ED, respectively). Two streams from dye–salt mixtures were received—one being enriched with dye alone (diluate) and the other one being a salt solution with no organic matter (concentrate).

Xue et al. [14] utilized the ED process to produce a salt-free dye. Acid Blue 9 (AB9) solution containing both mono- and divalent salts was subjected to ED desalination. The authors observed an uncommon monovalent ion selectivity—98% of chloride ions were eliminated from the feed. Simultaneously, nearly all divalent ions ${(SO}_{4}^{2-}$) were transported from the feed to the concentrate compartments. Finally, it was possible to receive a high dye concentration in the final product (200 g/L). Due to the high affinity between AB9 anions and the positively charged anion-exchange membranes (AMs), the dye particles were intensively deposited on the surface of the AMs. Fortunately, the desorption of the adsorbed AB9 dye was possible by applying a well-designed cleaning procedure. Targeting textile dye-bath desalination, Lafi et al. [15] performed laboratory ED experiments on the elimination of sodium chloride from Methylene Blue (MB) solution. The initial dye concentration was rather low (2–8.5 mg/L), while the initial NaCl concentration was typical for dye-baths and varied from 0.6 to 2.4 g NaCl/L. It was found that conventional ED enabled the efficient removal of NaCl from the salt–MB solution. The ions of the MB dye did not migrate through the IEMs to the concentrate compartments; nevertheless, the intensive adsorption of dye cations by the CM membranes was observed. This study confirmed that electrostatic attractions between the cations of the dyes and the negatively charged groups of the CM membranes can cause serious membrane contamination during the ED desalination of dye–salt solutions.

The fouling of IEMs caused by the charged dye molecules could be a main obstacle in the efficient valorisation of dye-containing effluents. Thus, various integrated membrane processes (involving ED) have been investigated recently. Lafi et al. [16] evaluated the performance of a hybrid ultrafiltration–electrodialysis (UF-ED) process while treating primary treated textile wastewater (PTWW). Ultrafiltration with ceramic membranes enabled the removal of organic matter, as well as colour reduction. Electrodialysis, in turn, was suitable for the removal of salt ions. The total dissolved solids (TDS) and the electrical conductivity of the PTWW (after UF treatment) were reduced by 94.2% and 97.1%, respectively. It was concluded that PTWW treated by the combined UF-ED process can be recycled as technological water. A new path in the fractionation of dye-house effluents was paved by Ye et al. [17]. They implemented a loose nanofiltration membrane (instead of a conventional anion-exchange membrane) in the electrodialytic stack. The loose NF membrane served as an anion-conductive membrane, and thus the fractionation of dye–salt mixtures was possible. The NF membrane with a molecular weight cut-off (MWCO) of 678 Da effectively retained reactive dyes with low molecular weight (MW) (<627 Da). At the same time, the NF membrane exhibited a high permeation for NaCl, and 98.9% desalination efficiency was obtained. It was concluded that the usage of the loose NF membrane in the ED stack (instead of the standard anion-exchange membrane) gave better results in reactive dye separation from the mineral salt in comparison to the standard ED process. Furthermore, due to the negative surface charge of the loose NF membrane, the intensity of membrane fouling was also reduced. Unfortunately, the performances of the proposed NF-based ED process when multivalent salts were present in the dye–salt mixtures were not validated. With a view to the recovery of dyes, salts, and water from high-salinity textile effluents, Lin et al. [18] proposed an innovative combined system of nanofiltration–diafiltration–electrodialysis (NF-DF-ED). The loose nanofiltration membrane with an MWCO of 800 Da enabled the satisfactory separation of reactive dyes from monovalent salt (NaCl). Owing to the large pore size of the NF membrane, NaCl salt freely permeated through the membrane, while reactive dyes were successfully rejected. Through the NF-DF concentration unit, the dye was concentrated from 2 to 17.9 g/L. The application of the ED unit in the next step enabled NaCl concentration up to 86 g/L with the simultaneous production of pure water, which was further reused in the NF-DF unit. It was concluded that the proposed integrated process (NF-DF-ED) had a capability to sustainably recover the resources (dyes, salts, and water) from textile effluents.

Based on this short literature review, it can be stated that the efficient fractionation of dye–salt mixtures is a great challenge. The NF process may seem the most appropriate one in the separation of reactive dyes from mineral salts; however, this process fails when multivalent salts are present in dye–salt mixtures. This drawback is an encouragement to search for suitable loose NF membranes. A few of the above-mentioned studies indicate that electromembrane processes have a potential for sustainable resources extraction from textile wastewater. However, the main drawback of the conventional ED process during the desalination of dye solutions is related to the strong adsorption of the charged dye molecules on the surface of IEMs. Therefore, a novel approach to the treatment of dye–salt aqueous solutions was proposed in this study. The monovalent anion-selective electrodialysis (MASED) process with standard cation-exchange membranes and monovalent selective anion-exchange membranes was applied to simultaneously recover reactive dyes and sodium chloride. It was anticipated that due to the usage of the MVA membranes, organic fouling would be markedly diminished. According to our knowledge, in the literature, there is generally a lack of papers dealing with the MASED process in the fractionation of reactive dye–salt mixtures, and thus the only premise to apply MASED was our previous positive results on the separation of direct dyes from mineral salts with the use of MASED [13]. Reactive dyes varying in MW and reactivity were chosen for this study. Generally, most of the common treatment methods are ineffective in the removal of reactive dyes from textile wastewater [19]. Thus, searching for treatment processes aiming at the recovery and reuse of reactive dyes is justified in view of the economical and environmental benefits.

## 2. Materials and Methods

### 2.1. Experimental Solutions

Model dye solutions containing mineral salt (sodium chloride) were used for the MASED process. Various reactive dyes (anionic in nature) were chosen for the experiments (Table 1). Two of them (RB5 and RO20) were bought from the Boruta Company (Bydgoszcz Poland), whereas three of them (RBB, RO16, and RR120) came from Merck Polska, Warszawa, Poland. All experimental solutions contained a single reactive dye (20, 50, or 100 mg/L) and NaCl (2, 4, or 6 g/L). The level of the applied dye concentration matched the dye content in the permeate after the UF pre-treatment of the exhausted dye-baths. It was assumed that the considered MASED process can be alternately preceded by the TUF process with reactive dye rejection by 80–97% [20].

### 2.2. Membranes and ED Stack

The laboratory ED system (PCCell BED-1, PCCell GmbH, Heusweiler, Germany) was used for the fractionation of model solutions containing reactive dye and salt. This system was provided with commercial ED membranes supplied from PCA GmbH (Heusweiler, Germany). As the reactive dyes were anionic in nature, to separate these dyes from monovalent mineral salt (NaCl) and reduce organic fouling, monovalent selective anion-exchange membranes (PC-MVA) and standard cation-exchange membranes (PC-SK) were chosen for the study. The ion-exchange membranes were purchased as a membrane set with flat spacers compatible with the PCCell Bed-1. The effective surface area of each membrane was 64 cm^2^. The description of the membranes is given in Table 2. The fresh IEMs were subjected to a cycling procedure with the use of 0.1 N HCl and 0.1 N NaOH solutions in the following sequence:

- Immersion in HCl for 2 h and then flushing with water for the Cl^−^ ions to disappear;
- Immersion in NaOH for 2 h and then flushing with water for the pH to decrease to <8.3;
- Immersion in HCl for 2 h and then flushing with water for the Cl^−^ ions to disappear.

The ED stack (PCCell 64002 model) contained 10 compartment pairs, each consisting of a diluate and a concentrate cell. Hence, 10 monovalent selective anion-exchange (PC-MVA) and 11 cation-exchange membranes (PC-SK) were put in the stack. Each cell had a thickness of 0.5 mm.

The diluate, concentrate, and electrode solutions circulated independently in the ED system with a flow rate of 10–100 L/h (with a preferable value of 90 L/h fixed at the beginning of MASED). The ED installation was provided with 3 tanks—1 internal tank for the electrode solution (9 L) and 2 external tanks for the diluate and concentrate solutions (2 L each). A DC power supply (with a maximum output voltage of 24 V and an amperage of 5 A) was the integrated part of the ED system.

**Table 2:** Description of the PCA IEMs (according to the PCCell ED 64002 manual).

Parameter	Membrane	
	Cation-Exchange PC-SK	Monovalent Selective Anion-Exchange PC-MVA
Producer	PCA GmbH (Germany)	
Thickness, µm	90–130	110
Ion-exchange capacity, mmol/g	Approx. 1.2	Approx. 1.0
Electrical resistance, Ω cm^2^	1–3	20
Thermal stability, °C	Maximal 60	Maximal 40
Chemical stability (pH range)	0–9	0–7
Burst strength, MPa	0.4	N/A
Permselectivity (transfer number)		
t-K^+^	>0.95	
t-Cl^−^		>0.97
Ionic form	Na^+^	Cl^−^
Functional group	${-SO}_{3}^{-}$	${-NR}_{3}^{+}$

### 2.3. Methodology

Prior to the main experiments involving the desalination of reactive dye solutions by MASED (monovalent anion-selective ED), the desalination of water–salt solutions (2, 4, 6, and 10 g NaCl/L) was performed for comparison purposes.

In the course of the MASED experiments, it was assumed that the final volumes of the diluate and concentrate did not change markedly in relation to their initial volumes (2 L). At the beginning of the process, the initial salt amount in the diluate and concentrate loops was the same. The right amounts of reactive dyes contained merely the diluate feed solutions. In the first stage of the experiments, the starting dye concentration in the diluate feed was variable (20, 50, and 100 mg/L), whereas the NaCl concentration in the dye solutions was constant (2 g/L). In the experiments’ second stage, the tested solutions contained 20 mg/L of dye, while the salt concentration was variable (2, 4, and 6 g/L). NaCl solution (0.05 mol/L) was used as an electrode rinsing liquid.

The MASED system operated in a batch mode, i.e., both the diluate and the concentrate circulated in the stack until the fractionation of dye–salt solutions was achieved. It was necessary to monitor the voltage between the electrodes continuously, as an increase in voltage > 24 V would be a reason to finish MASED instantly. The first experimental MASED series with dye solutions containing 2 g NaCl/L was performed at a constant current intensity equal to 0.15 A (which corresponded to a current density of 2.35 mA/cm^2^). The second experimental MASED series with variable salt concentration (2–10 g/L) was performed under a constant current intensity range from 0.15 to 0.75 A (from 2.35 to 11.75 mA/cm^2^, respectively). The range of the experimental current density was adjusted on the basis of the limiting current evaluation (Equation (3)) as well as on the results of our previous study [13,21].

A simple cleaning procedure (with distilled water) of the ED installation was employed when around a 20% drop in the flow rate of the diluate/concentrate solutions was observed. The cleaning procedure was also applied after the completion of the ED experiments with a given dye. This cleaning action enabled the flow to be restored, as well as being helpful in maintaining ion flux in the course of MASED.

During the MASED process, the quality of both diluate and concentrate streams was verified every 10 min. The concentration of organic dyes was determined by absorbance measurements with the use of the spectrophotometer UV-Vis U-1900 (Hitachi, Tokyo, Japan). The salt content was estimated indirectly by measuring the electrical conductivity of the diluate and concentrate samples. A conductivity meter, Elmetron CC-411, was used for this analysis. As the conductivity of dye–salt mixtures was a few orders higher than the conductivity of model dye solutions (without salt), the applied method of salt amount determination was affected by a minor error (<0.8%).

The measurements were triplicated in the course of the experiments.

The dye retention (R_d_, %) in the diluate cell, i.e., the share of the starting dye amount that remained in the diluate, was calculated with the use of Equation (1):

(1) $$R_{d}=\frac{C_{td}}{C_{id}}\times 100\%$$

where

C_td_—the actual dye concentration in the diluate (mg/L);
C_id_—the initial dye concentration in the diluate (mg/L).

The desalination efficiency (R_s_, %) was calculated using Equation (2):

(2) $$R_{s}=\frac{C_{i}-C_{s}}{C_{i}}\times 100\%$$

where

C_s_—the actual diluate conductivity (mS/cm);
C_i_—the initial diluate conductivity (mS/cm).

The limiting current density was estimated theoretically according to the simplified equation delivered by Rauntenbach et al. [22]:

(3) $$i_{lim}=\frac{k\times C_{D}^{+}\times F}{T_{m}^{+}-T^{+}}$$

where

$i_{lim}$—the limiting current density (A/m^2^);
k—the mass transfer coefficient (L/m^2^ s);
$C_{D}^{+}$—the concentration of cations (Na^+^) in the diluate after desalination (eq/L);
F—the Faraday constant (96,500 As/eq);
$T_{m}^{+}$—the transfer number of cations in the membrane (0.95);
$T^{+}$—the transfer number of cations in the solution (0.45).

It was assumed that the final salt concentration in the diluate should not be higher than 5–10% of the salt concentration in the diluate at the beginning of MASED.

The electrical energy consumption (EC) and the specific electrical energy consumption (E_v_) were calculated according to the following equations:

(4) $$EC=I\int_{0}^{t}Udt$$

(5) $$E_{v}=\frac{EC}{V_{d}}$$

where

EC—the energy demand for the MASED process (kWh);
I—the current intensity (A);
U—the voltage (V);
t—the time of the process (h);
E_V_—the specific electrical energy consumption (kWh/m^3^);
V_d_—the volume of the treated solution (diluate, 2 L).

Equation (4) only involves the energy used for ion transport across the IEMs. The energy needed to pump the diluate and concentrate solutions through the ED stack was not taken into account.

The dye mass balance was made with the aim of evaluating dye vulnerability for adsorption on the IEMs and in the ED system. The mass of the accumulated dye was calculated according to Equation (6):

(6) $$M=\left(V_{id}C_{id}+V_{ic}C_{ic}\right)-(V_{fd}C_{fd}+V_{fc}C_{fc})$$

where

M—the mass of the dye accumulated on/in the IEMs (mg);
V_id_, V_fd_—the initial and final volume of the diluate, respectively (2 L);
V_ic_, V_fc_—the initial and final volume of the concentrate, respectively (2 L);
C_id_, C_fd_—the initial and final concentration of the dye in the diluate, respectively (mg/L);
C_ic_, C_fc_—the initial and final concentration of the dye in the concentrate, respectively (mg/L).

## 3. Results and Discussion

### 3.1. Fractionation of Binary Mixtures Containing Dye and Salt by MASED

The experiments were focused on the effective separation of salt from the dye–salt mixture. The effect of dye concentration as well as salt concentration in the dye–salt mixtures on process efficiency was evaluated. The MASED process was performed at a current intensity of 0.15, 0.3, 0.45, and 0.75 A (which corresponded to a current density of 2.35, 4.70, 7.05, and 11.75 mA/cm^2^, respectively). The range of the applied electrical current was chosen on the basis of the limiting current density, which was established theoretically [22]. The limiting current density was calculated for a salt concentration of 2 g NaCl/L, according to Equation (3). Depending on the assumed desalination efficiency of the sodium chloride solution (90 or 95%), it was equal to 6.56 or 3.28 mA/cm^2^, respectively. In practice, it is desirable not to exceed the current density above a value of 0.8$i_{lim}$. The chosen value of the current density (2.35 mA/cm^2^ for the solution containing 2 g NaCl/L) met the above condition, and thus high desalination efficiency could be expected. For the experiments with the increased salt amount (4, 6, and 10 g/L), the limiting current density was established approximately by taking into account Faraday’s law and the value of the limiting current density for a salt concentration of 2 g/L (3.28 mA/cm^2^).

**Figure 1:**
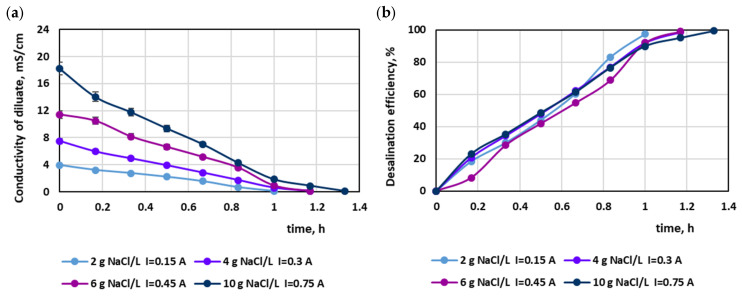
Desalination of NaCl solutions by monovalent anion-selective electrodialysis (MASED): (**a**) diluate electrical conductivity versus operation time and (**b**) desalination efficiency versus operation time.

In the preliminary tests, the MASED process was evaluated in relation to the variable salt concentration in the aqueous solutions (with no dye). Thus, a current intensity of 0.15, 0.3, 0.45, and 0.75 A (which corresponded to 2.35, 4.7, 7.05, and 11.75 mA/cm^2^) was applied for salt concentrations equal to 2, 4, 6, and 10 g NaCl/L, respectively. The effect of the salt content in the treated solution on MASED efficiency is shown in Figure 1. The obtained desalination efficiency at a constant current density was really high (97.4–99.4%). The highest desalination efficiency was noted for the highest salt concentration (10 g/L); however, in this case, the longest operational time was needed. It can be seen that Faraday’s law did not quite work—although the electrical current was directly proportional to the salt content, the process time was elongated by approximately 20–33% with increasing salt content (in comparison to the MASED time for a NaCl concentration of 2 g NaCl/L). This finding can be attributed to the worsening of the conditions of ion-selective migration, due to the increased concentration gradient between the concentrate and diluate cells. The current efficiencies evaluated according to the method described by Hyder et al. [23] confirmed the adverse conditions for ion migration under an elevated salt concentration. The current efficiency for the desalination of solutions containing 2, 4, and 6 g NaCl/L amounted to 110%, 91.8%, and 92.4%, respectively, whereas for the desalination of the solution containing 10 g NaCl/L, a markedly lower current efficiency was noted (78.4%), indicating that an undesired phenomenon such as the back diffusion of salt ions from the concentrate to the diluate chamber was enhanced. Hyder et al. [23] and Wei et al. [24] also observed a decreasing trend in current efficiency with increasing feed salinity. The final diluate electrical conductivity was comparable for all NaCl concentrations and amounted to 0.1–0.11 mS/cm. The highest final conductivity was observed for the highest salt concentration (10 g/L).

**Figure 2:**
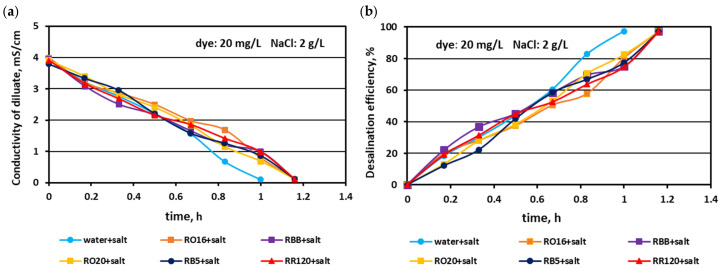
Desalination of dye–salt mixtures by MASED for various reactive dyes (20 mg/L): (**a**) diluate electrical conductivity versus operation time and (**b**) desalination efficiency versus operation time; current: 0.15 A (2.35 mA/cm^2^).

**Figure 3:**
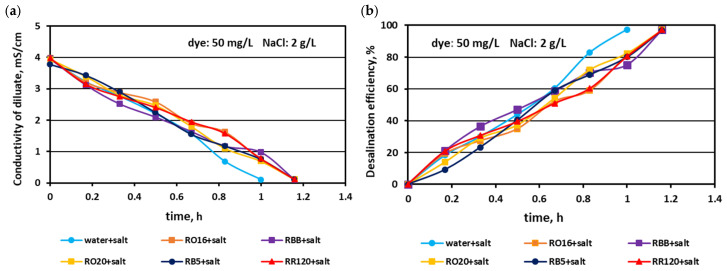
Desalination of dye–salt mixtures by MASED for various reactive dyes (50 mg/L): (**a**) diluate electrical conductivity versus operation time and (**b**) desalination efficiency versus operation time; current: 0.15 A (2.35 mA/cm^2^).

**Figure 4:**
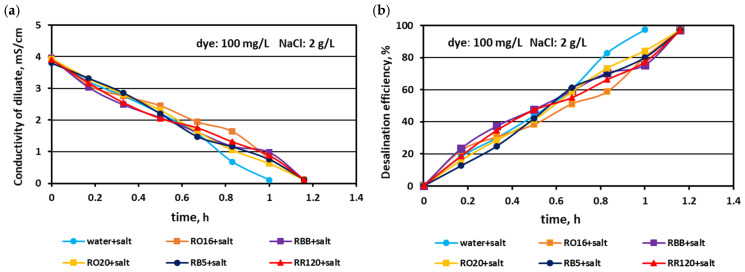
Desalination of dye–salt mixtures by MASED for various reactive dyes (100 mg/L): (**a**) diluate electrical conductivity versus operation time and (**b**) desalination efficiency versus operation time; current: 0.15 A (2.35 mA/cm^2^).

In the first experimental stage, the MASED performances were verified for binary solutions containing dye and NaCl. The concentration of the reactive dyes varied (from 20 to 100 mg/L), whereas the salt content in the dye solutions was constant (2 g NaCl/L). The effect of dye amount on desalination efficacy for the various dyes used in the study is given in Figure 2, Figure 3 and Figure 4. Based on these figures, it was stated that the salt recovery was very good and amounted to 97.1–97.3%, irrespectively of the initial dye concentration and dye type. The diluate conductivity at the end of MASED was around 0.11–0.15 mS/cm. The desalination results obtained for the monovalent anion-selective ED (MASED) were slightly worse than the results received for the standard ED [21], probably due to the improved surface compactness of the PC-MVA membrane in comparison with the surface compactness of the standard anion-exchange membrane (PC-SA). The desalination time for the dye–salt mixtures amounted to 70 min, irrespectively of the initial dye concentration and dye type, and was 10 min longer than the operational time needed for the desalination of the NaCl solution (with no dye). These observations confirmed the inhibition effect of the dye’s presence on salt ion migration. The shape of the obtained relationships (i.e., the conductivity of diluate and the efficiency of salt removal versus time) were similar, irrespectively of the used dye. This finding was in line with expectations since the salt concentration in the dye solutions was constant (2 g NaCl/L). As has already been mentioned, the operational time of the electrodialysis depends on the initial salt concentration (according to Faraday’s law), and the impact of dye type and dye concentration on the ED course should be negligible.

**Figure 5:**
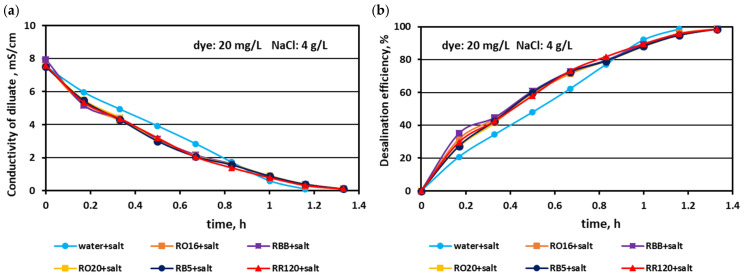
Desalination of dye–salt mixtures by MASED for various reactive dyes (20 mg/L): (**a**) diluate electrical conductivity versus operation time and (**b**) desalination efficiency versus operation time; current: 0.3 A (4.70 mA/cm^2^).

**Figure 6:**
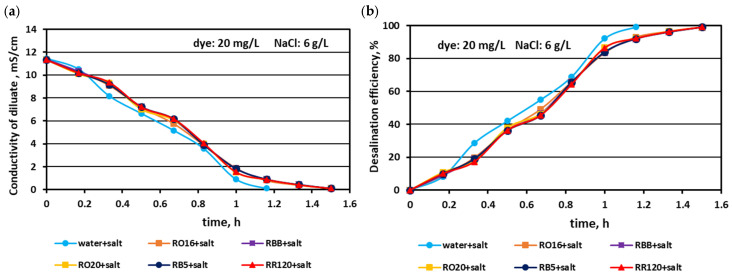
Desalination of dye–salt mixtures by MASED for various reactive dyes (20 mg/L): (**a**) diluate electrical conductivity versus operation time and (**b**) desalination efficiency versus operation time; current: 0.45 A (7.05 mA/cm^2^).

The effect of the elevated salt amount (4 and 6 g NaCl/L) on the efficiency of salt removal from solutions containing various reactive dyes (20 mg/L) is given in Figure 5 and Figure 6. Generally, the MASED desalination effect was extremely good, irrespectively of salt concentration and the type of reactive dye present in the treated solution. Only minor differences were observed in salt recovery depending on salt content. With increasing salt content, the desalination efficiency slightly improved from 97% (at 2 g NaCl/L; Figure 2) to around 98.5% for the solution with 4 g NaCl/L (Figure 5), and to almost 99% for the solution with 6 g NaCl/L (Figure 6). The final electrical conductivity of the diluate slightly worsened with the increase in salt concentration and was equal to 0.105–0.11 mS/cm (for 2 g NaCl/L), 0.11–0.112 mS/cm (for 4 g NaCl/L), and 0.116–0.119 mS/cm (for 6 g NaCl/L). The obvious effect of the variable salt concentration on MASED performance was the elongation of the operation time with increasing salt content. The desalination time increased from 70 min at 2 g NaCl/L to 90 min at 6 g NaCl/L. The desalination time for the dye–salt mixtures was 10–20 min longer than the operational time needed for the desalination of the NaCl solution (with no dye), although the applied current was chosen to be directly proportional to the salt concentration (according to Faraday’s law). Again, these observations confirmed the inhibition effect of the dye’s presence on the kinetics of ion migration through the IEMs. This negative impact of the dye molecules on the desalination rate was especially pronounced at the end of MASED (Figure 5 and Figure 6). Approximately after 1 h of operation, the desalination kinetics of the solutions containing 4 and 6 g NaCl/L was diminished. It can be supposed that the PC-MVA membranes were covered with a dense dye-fouling layer and the restriction on transmembrane ion migration can be observed. Similarly, as for the first experimental stage, the shapes of the obtained correlations (i.e., the variation in the diluate conductivity and desalination efficacy versus operational time) were almost identical, regardless of the tested dye.

### 3.2. Reactive Dye Retention in the Course of the MASED Process

To meet the rules of the circular economy, besides effective salt separation from the exhausted dye-baths, the purpose of this research equally involved the recovery of the reactive dyes. For this reason, the dye particles should be retained in the diluate compartments. Furthermore, it is desired to receive the final diluate solutions containing reactive dyes at concentrations close to the feed dye concentration. Thus, the monitoring of the dye concentration in the diluate and concentrate cells in the course of MASED was worth investigating. It was expected that the monovalent selective anion-exchange membranes (MVA membranes) would be an effective barrier for the unwanted migration of dye particles from the diluate cells to the concentrate cells.

#### 3.2.1. Dye Reactivity and Susceptibility to Adsorption by Ion-Exchange Membranes

One of the features of the reactive dyes is their various reactivity resulting from possessing different reactive groups in dye molecules, such as monochlorotriazine, monofluorotriazine, dichlorotriazine, dichloroquinoxaline, trichloropyrimidine, and vinyl sulfone [25]. These reactive groups are capable of reacting with textile fibres during the dyeing process, as well as with membrane polymer in the course of electrodialysis. The dichlorotriazine groups are the most reactive. The dyes of medium reactivity contain the vinyl sulfone groups. Finally, the monochlorotriazine groups have the lowest reactivity [25]. Therefore, the reactive dye behaviour during MASED should be analyzed in view of their reactivity. A similar approach was applied by Won et al. [26] during the analysis of reactive dye adsorption by protonated waste biomass. They found that at a solution pH > 7, the binding mechanism of reactive dyes by the biomass occurred according to their reactivity. The reactive dye biosorption on the biomass was the effect of a chemical reaction between the hydroxyl groups of the waste biomass and the dye reactive groups. In the course of MASED, the pH of the diluate solutions was mostly > 7, and thus it can be anticipated that the dye reactivity has a great impact on their adsorption by the IEMs.

#### 3.2.2. Effect of Feed Dye Concentration on Dye Adsorption by PC-MVA Membranes

**Figure 7:**
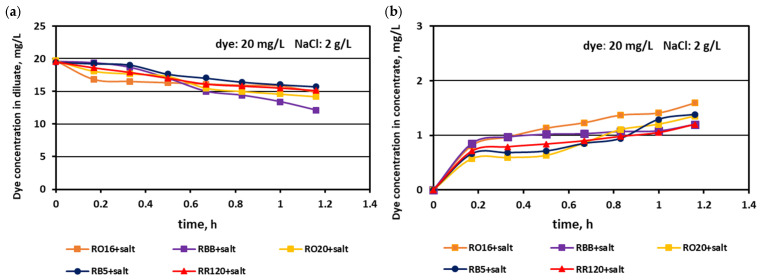
Desalination of dye–salt mixtures by MASED for various reactive dyes (20 mg/L): (**a**) dye concentration in diluate versus operation time and (**b**) dye concentration in concentrate versus operation time; current: 0.15 A (2.35 mA/cm^2^).

**Figure 8:**
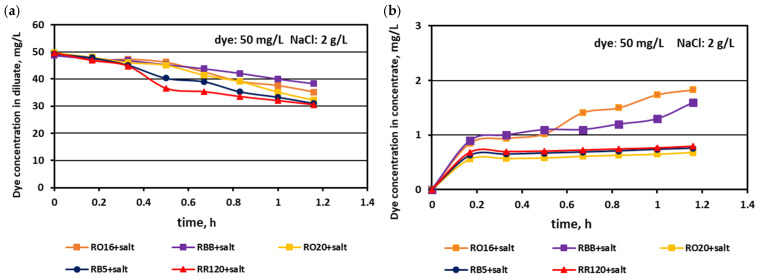
Desalination of dye–salt mixtures by MASED for various reactive dyes (50 mg/L): (**a**) dye concentration in diluate versus operation time and (**b**) dye concentration in concentrate versus operation time; current: 0.15 A (2.35 mA/cm^2^).

**Figure 9:**
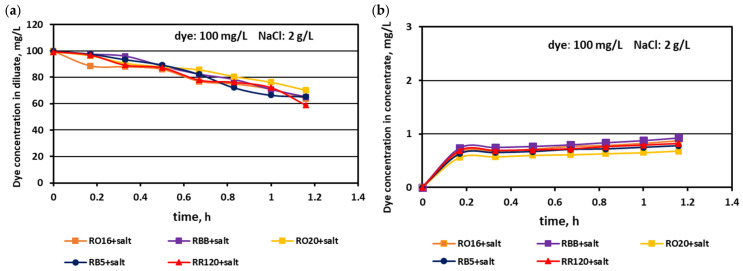
Desalination of dye–salt mixtures by MASED for various reactive dyes (100 mg/L): (**a**) dye concentration in diluate versus operation time and (**b**) dye concentration in concentrate versus operation time; current: 0.15 A (2.35 mA/cm^2^).

The relationship between the actual dye concentration in both the diluate and concentrate compartments versus the operational time for various dye concentrations at the beginning of MASED is given in Figure 7, Figure 8 and Figure 9. Contrary to expectations, intensive dye adsorption in the course of MASED with PC-MVA membranes was observed for all dye solutions. The results given in Figure 7, Figure 8 and Figure 9 indicated a significant drop in dye concentration in the diluate cells with process time.

The highest decrease in the diluate dye concentration with process time was observed for the RR120 dye (at concentrations of 50 and 100 mg/L), which had the highest molecular weight (1469.98 Da) among all tested dyes and the highest adsorption potential, resulting from possessing two of the most reactive groups (dichlorotriazine groups) in the dye molecule (Table 1). On the other hand, the RBB dye (molecular weight: 636.53 Da) also showed a high decrease in its concentration in the diluate with operation time (Figure 7a), which can rather be attributed to the small size and spherical shape of the dye molecule [25] than to the molecule reactivity (only one vinyl sulfone reactive group of moderate reactivity). According to the results shown in Figure 7b, Figure 8b and Figure 9b, generally, there was a minimal transport of dye molecules from the diluate to the concentrate cells. However, a low steady amount of dye in the concentrate cells (below 1 mg/L) was kept merely when the feed dye concentration was equal to 100 mg/L. In the case of lower initial dye concentrations (20 and 50 mg/L), continuous dye migration from the diluate to the concentrate cells was observed, and the final dye amount in concentrate cells reached around 1.6–1.8 mg/L. Such dye behaviour was especially noticeable for low-molecular-weight dyes such as RO16 and RBB. In common with the standard ED experiments [21], the continuous decrease in dye amount in the diluate compartments, with simultaneously rather small dye movement into the concentrate compartments, confirms the dye adsorption phenomenon even in the case of the monovalent selective anion-exchange membranes (acting together with the standard cation exchange-membranes). This phenomenon confirmed the importance of dye reactivity on the final result of dye–salt fractionation by ED.

**Figure 10:**
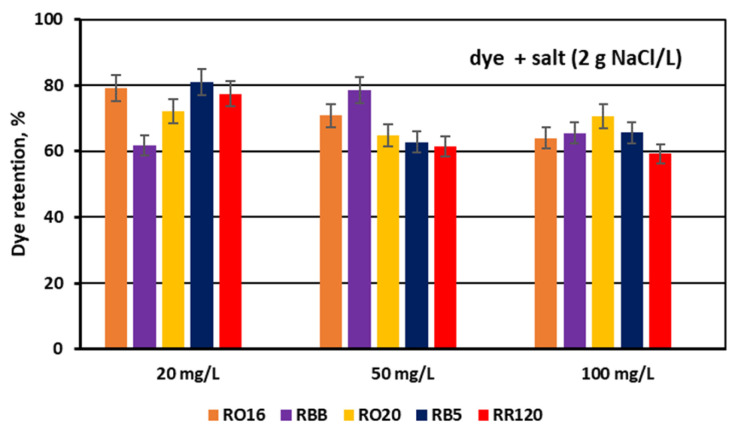
Dye retention in diluate compartments for various reactive dyes at variable dye concentration (20, 50, and 100 mg/L); current: 0.15 A (2.35 mA/cm^2^).

**Figure 11:**
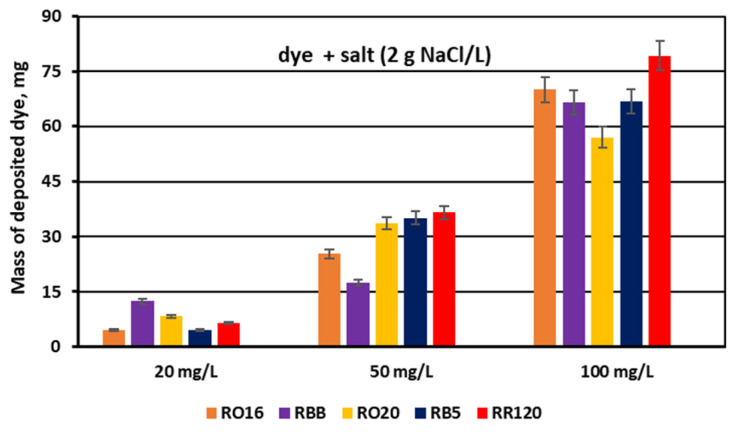
Mass of dye deposited on/in PC-MVA membranes for various reactive dyes at variable dye concentration (20, 50, and 100 mg/L); current: 0.15 A (2.35 mA/cm^2^).

The dye adsorption issue in the course of monovalent anion-selective ED (MASED) can be evaluated in detail through the dye retention coefficient (Figure 10) as well as by the deposited dye mass (Figure 11). As presented in Figure 10, dye retention in the diluate cells after the end of MASED varied from 59 to 81%, and this parameter was slightly worse than that noted in the course of standard ED (68–91%) [21]. In the case of the MASED process, the lowest retention was observed not only for the low-molecular-weight dye (the RBB dye at a feed concentration of 20 mg/L), but also for high-molecular-weight-dyes such as RB5 and RR120 (at feed concentrations of 50 and 100 mg/L). No direct correlation between the initial dye concentration and dye retention was found, but generally, it was noted that the highest feed dye concentration resulted in the lowest dye retention. This observation can be explained by a strong tendency of the reactive dyes to self-associate [27]. With the increase in dye concentration, the intensity of aggregation will be enhanced, more dye aggregates will be deposited on the ion-exchange membrane surface, and dye retention in the diluate cells will be lowered. It should also be underlined that dye self-association is related to dye solubility, which, in turn, depends on the various solubilizing groups present in the reactive dye molecules. Thus, it was rather difficult to find a univocal relationship between dye retention, dye type, and dye concentration. Instead, an evident correlation between the initial dye concentration and the mass of dye deposited in the ED stack was confirmed—a high initial dye concentration was accompanied by a high dye amount adsorbed by the ion-exchange membranes (Figure 11). The share of the accumulated dye mass fluctuated from 12 to even 40% (in the case of the RR120 dye) of the total dye amount in the diluate loop at the beginning of MASED. Surprisingly, in the case of the standard ED process, this percentage of deposited dye mass was lower (6–32%) [21]. It seems that the implementation of the monovalent selective anion-exchange membranes did not give the expected results, i.e., the limitation of dye adsorption by the membranes, and even enhanced the interactions between the dye particles and the ion-exchange membranes, especially for the high-molecular-weight dyes characterized by the highest reactivity (e.g., the RB5 and RR120 dyes). As has already been proved, the RR120 dye was especially prone to build up on the membrane surface. This dye was strongly adsorbed by the membranes, and the accumulated mass of the RR120 dye constituted even 16–40% of the total dye mass in the diluate cells at the beginning of MASED. Again, it seems that this high uptake of the reactive dye particles by the IEMs could be a challenge in a successful valorisation of the exhausted dye-baths in the course of electrodialysis.

#### 3.2.3. Effect of Feed Salt Concentration on Dye Adsorption by PC-MVA Membranes

**Figure 12:**
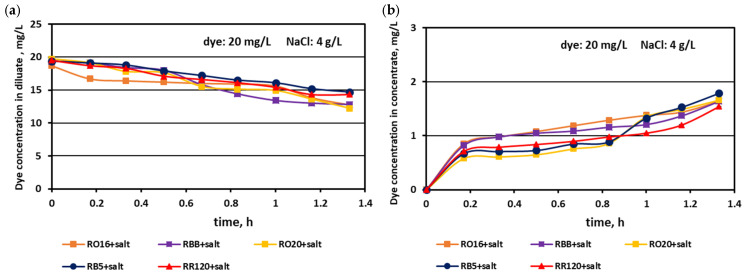
Desalination of dye–salt mixtures by MASED for various reactive dyes (20 mg/L): (**a**) dye concentration in diluate versus operation time and (**b**) dye concentration in concentrate versus operation time; current: 0.3 A (4.7 mA/cm^2^).

**Figure 13:**
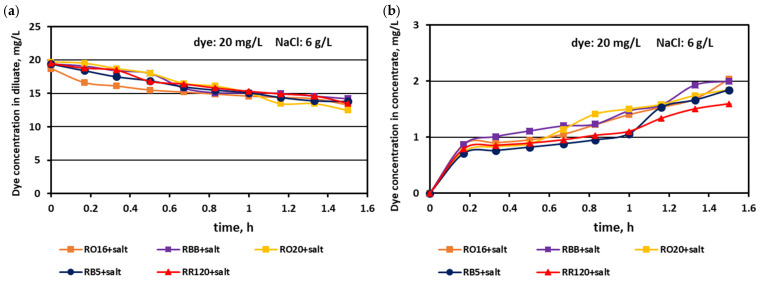
Desalination of dye–salt mixtures by MASED for various reactive dyes (20 mg/L): (**a**) dye concentration in diluate versus operation time and (**b**) dye concentration in concentrate versus operation time; current: 0.45 A (7.05 mA/cm^2^).

It has already been underlined that the most important consideration in the valorisation of dye–salt mixtures is a high dye recovery. As has been stated above, due to their high reactivity, reactive dyes are strongly susceptible to adsorption by polymeric membranes. On the other hand, dye behaviour during the ED process is the effect of interactions between dye anionic macro-particles, salt ions, and membrane material. Thus, the way that the dyes behaved throughout MASED under an elevated salt content was worth investigating. The effect of the elevated salt content on dye concentration in the diluate and concentrate cells in the course of MASED is given in Figure 12 and Figure 13.

Generally, a systematic decrease in dye amount in the diluate compartments with a simultaneous increase in dye concentration in the concentrate compartments with time was observed, irrespectively of the salt concentration in the treated solutions. These relationships were slightly more pronounced with increasing salt concentration and decreasing dye molecular weight. The highest decrease in dye concentration in the diluate compartments was noted for the RO16, RBB, and RO20 dyes, which had the lowest molecular weights. The movement of dye particles from the diluate to the concentrate cells was minor, and the final dye amount in the concentrate did not exceed 2 mg/L. However, the final content of the dyes in the concentrate cells slightly increased with increasing salt concentration, especially in the case of low-molecular-weight dyes (RO16 and RBB). Such dye behaviour is somewhat contradictory to the previous results describing the intensive adsorption potential of the high-molecular-weight dye (RR120) due to the existence of two strongly reactive groups in the dye particle (at a salt concentration of 2 g/L). It is well known that mineral salts are used to increase the affinity of reactive dyes to textile fibres during the dyeing process [3]. Taking into account the above fact, it becomes clearer that with increasing NaCl concentration in the dye–salt mixture, the potential of dye adsorption on and/or in the polymeric membranes also increases. Furthermore, the low-molecular-weight dyes (RO16 and RBB) could more easily migrate through the IEMs, as due to the higher electrical conductivity, the electric current induced was also higher (in comparison to the current at a low salt concentration) [15].

**Figure 14:**
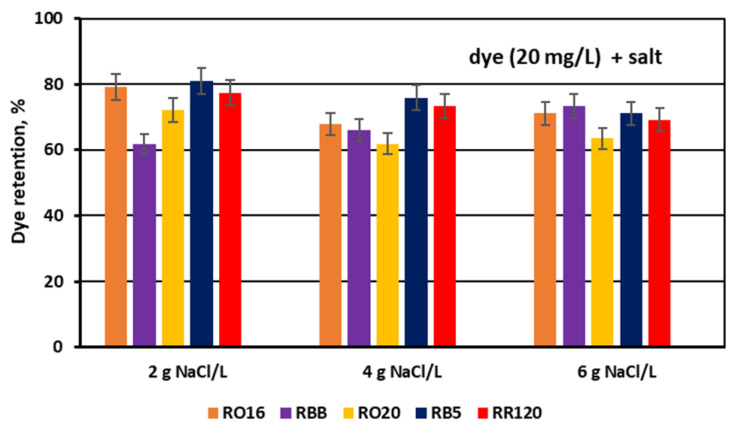
Dye retention in diluate compartments for various reactive dyes at variable NaCl concentration (2, 4, and 6 g NaCl/L); current: 0.15, 0.3, and 0.45 A, respectively.

**Figure 15:**
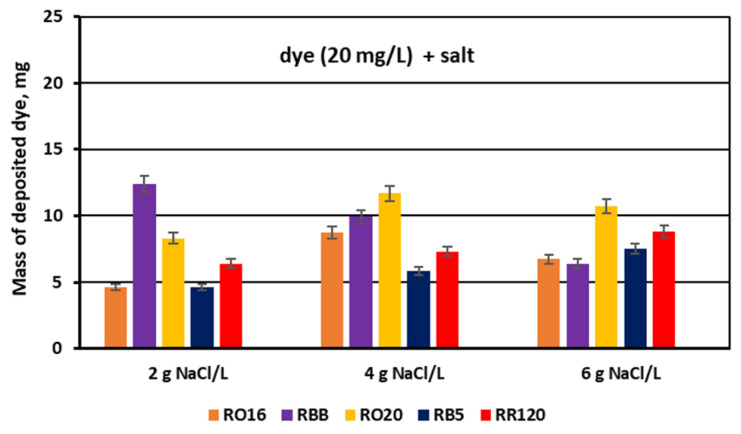
Mass of dye deposited on/in the PC-MVA membranes for various reactive dyes at variable NaCl concentration (2, 4, and 6 g NaCl/L); current: 0.15, 0.3, and 0.45 A, respectively.

For the experimental series with an elevated salt concentration, the above suppositions were also confirmed by the determined dye retention coefficients (Figure 14) along with the calculated masses of the deposited dyes (Figure 15).

As presented in Figure 14, dye retention in the diluate cells after the end of the MASED process varied from 61.7 to 80.9% at a salt concentration of 2 g NaCl/L, from 61.9 to 75.8% at a salt concentration of 4 g NaCl/L, and from 63.5 to 73.2% at a salt concentration of 6 g NaCl/L. Taking into account the insignificant migration of dye particles to the concentrate cells, it can be concluded that the elevated salt concentration in the dye–salt mixtures brought about the intensification of dye adsorption in the ED stack. The possible explanation of this finding could be related to the increasing dye surface negative charge with increasing salt concentration in the dye–salt mixtures (an increasing NaCl concentration results in increasing solution pH) [15]. The elevated surface negative charge of the reactive dyes resulted in a stronger electrostatic interaction between the dye particles and the PC-MVA membrane. However, in the case of the RBB dye, the opposite phenomenon was observed; i.e., its retention in the diluate cells increased with increasing salt concentration. The mechanism of such behaviour of the RBB dye is not clear—it is possible that the salt shielding effect on this dye is dominant (due to its low reactivity), allowing a diminished interaction with the PC-MVA membrane.

The masses of dyes deposited in/on the MVA membranes (Figure 15) are the reflection of the calculated dye retention coefficients. The share of the adsorbed dye mass fluctuated from 11 to even 30% (in the case of the RRB dye) in relation to the total dye amount in the diluate cells at the beginning of MASED. As has already been proved, the RR120 dye showed high vulnerability to adsorption by the IEMs, but this observation was valid for the increased feed dye concentration (50 and 120 mg/L) (Figure 10 and Figure 11). In the analyzed case, i.e., for the lowest dye concentration (20 mg/L), the RBB dye exhibited the highest adsorption potential for the low salt concentration (2 g NaCl/L). Although this dye possesses only one functional group of moderate reactivity, its particle has a shape close to a sphere with a diameter of around 2.02 nm [28]. Thus, the migration of this dye into the membrane matrix could be facilitated.

It seems that the high salt content in the dye–salt mixtures with the simultaneous high chemical reactivity of the dyes could be a barrier to the successful valorisation of the exhausted dye-baths, i.e., the recovery of the reactive dyes without any mass loss in the course of the electrodialysis. Nevertheless, the monovalent selective anion-exchange membranes can be easily applied in the treatment of textile effluents containing less reactive dyes. It was reported [13] that the usage of the PC-MVA membranes in the ED process for the separation of mineral salts from solutions containing various direct dyes resulted in the almost complete recovery of the dyes. On the basis of MVA membranes, new membranes with antifouling properties were developed. These new ion-exchange membranes have additional electric-charged layers on their surface with a charge opposite to the charge of the fixed groups in the membrane matrix. It was proved that these membranes did not show organic fouling caused by either aromatic or aliphatic substances due to the electrostatic interaction between the organic anions and the charged groups of the additional thin layer on the membrane surface [29,30]. Therefore, to further promote the MVA membranes, it is essential to modify them and develop new membranes with antifouling properties and monovalent anion selectivity.

### 3.3. Power Demand for Desalination of Dye–Salt Mixtures by MASED

**Figure 16:**
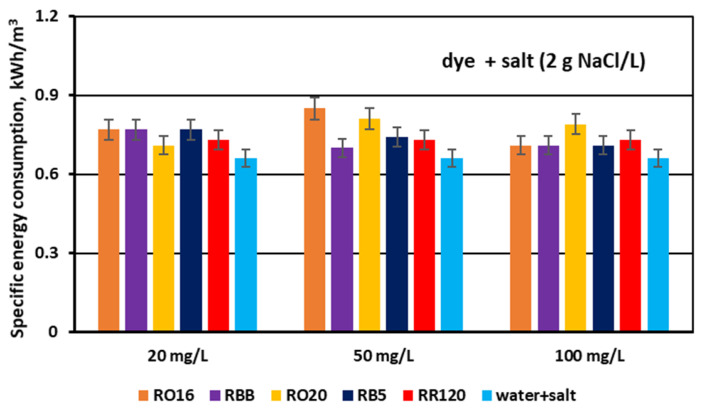
Specific electrical energy consumption during desalination of dye–salt mixtures by MASED for various reactive dyes at variable dye concentration (20, 50, and 100 mg/L); current: 0.15 A (2.35 mA/cm^2^).

**Figure 17:**
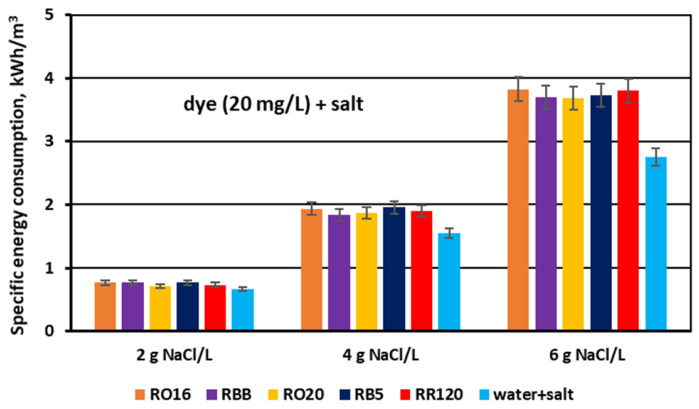
Specific electrical energy consumption during desalination of dye–salt mixtures by MASED for various reactive dyes at variable NaCl concentration (2, 4, and 6 g/L); current: 0.15; 0.3 and 0.45 A, respectively.

The specific electrical energy consumption (E_v_) needed for the desalination of the dye–salt mixtures by MASED was determined and presented in Figure 16 and Figure 17. For the first stage of the experiments performed at a constant NaCl concentration (2 g/L), the calculated E_v_ values fluctuated slightly (from 0.7 to 0.85 kWh/m^3^). As the salt concentration was the same and the applied current was constant (0.15 A) in the course of MASED, it is obvious that the variation in energy consumption was due to the presence of the reactive dyes in the treated solutions. Reactive dyes can influence salt ion transport across the ion-exchange membranes mainly by impeding ion migration from the diluate to the concentrate cells. Thus, the energy demand for salt removal from the binary solutions (containing both salt and dye) was noticeably greater than the energy consumption required for the desalination of the NaCl solution (with no dye) (Figure 16). As there is a slight differentiation in power consumption depending on dye type and dye concentration (at a constant salt concentration), some other phenomena, influencing dye and salt behaviour during the MASED process, should be considered. The movement of salt ions through the IEMs may also be influenced by the shielding effect of the salt on the reactive dye, and consequently, by the varied intensity of the interactions between the dyes and the membranes. The electrostatic attractions between the anions of the dye molecules and the MVA membrane may result in the formation of a negatively charged membrane layer (due to the negative surface charge of the dye molecules) [31]. This layer can also disturb ion migration from the diluate to the concentrate cells. Presumably, the above-mentioned phenomena can be the reason for the slight differentiation in the specific energy consumption when the dye concentration in the tested solution was variable.

According to the results given in Figure 16, the energy consumption necessary for the desalination of the NaCl solution (2 g/L) in the course of MASED amounted to 0.66 kWh/m^3^. This E_v_ value, however, was slightly higher (around 5–8%) than the energy consumption noted for a similar process conducted with standard anion-exchange membranes (PC-SA) [21]. This observation seems reasonable as the electrical resistance of the PC-MVA membrane is higher than the electrical resistance of the PC-SA membrane (20 and 1–3 Ω cm^2^, respectively).

Finally, the effect of salt content on the specific electrical energy consumption (E_v_) needed for salt removal from dye–salt solutions was calculated and is presented in Figure 17. For comparison purposes, the results obtained for the aqueous salt solutions (no dye) are also included in this figure. The calculated E_v_ values varied markedly with increasing NaCl concentration and amounted to 0.71–0.77 kWh/m^3^ for a NaCl concentration of 2 g/L, 1.84–1.96 kWh/m^3^ for a NaCl concentration of 4 g/L, and 3.68–3.83 kWh/m^3^ for a NaCl concentration of 6 g/L. The given energy demand values are in accordance with Faraday’s law, as they are in direct proportion to the salt content and the used current intensity. Some minor fluctuations in power consumption (for a given salt content) could be the result of different conditions of mass transfer through the IEMs depending on dye type. As has already been discussed, the presence of the reactive dyes in the solutions subjected to MASED can influence salt ion transport due to some interactions between the dye particles, salt ions, and IEMs.

The amount of energy required for the fractionation of two-component solutions (containing both dye and salt) was higher than the power consumption needed for salt removal from one-component solutions (with no dye). This difference in power consumption should be assigned to the obstructed ion transport, as a result of the existence of dye particles in the diluate cells as well as in the MVA membranes (dye deposition). Subsequently, the energy consumption for the MASED fractionation of dye–salt mixtures rose by 10–30% in relation to the energy consumption determined for the MASED of aqueous solutions containing only NaCl.

The organic fouling of anion-exchange membranes has been observed by many researchers. Lee et al. [32] found that organic foulants characterized by a high negative charge can significantly affect ED performance, causing even irreversible fouling. The electrical resistance of anion-exchange membranes fouled by anionic surfactants increased by around 20% (in comparison to the electrical resistance of fresh membranes). This rise in membrane electrical resistance was accompanied by a comparable increase in energy consumption, i.e., by around 20%, in comparison with the energy consumption determined for the ED desalination of salt solutions free of foulants. Similarly, Zhao et al. [33] observed an extremely high increase in membrane resistance (from 2.5 Ω cm^2^ for fresh membranes to even 120–140 Ω cm^2^ for fouled membranes) due to the presence of anionic surfactants in the NaCl solution treated by the ED process. It was also underlined that small aliphatic organic compounds had a lower contribution to fouling than larger aromatic organics due to electrostatic interactions. More recently, Phukan et al. [34] used three complex industrial streams (corn steep liquor, citric acid by-product, and cheese whey) to evaluate the mechanism and fouling intensity of commercial anion-exchange membranes. Around 10–30% increase in membrane electrical resistance after the ED process (in comparison to the electrical resistance of the pristine ion-exchange membranes) was observed. This study also revealed that low-molecular-weight organic fractions entered into the membrane matrix, whereas humic substances, due to their high molecular weight, were adsorbed on the membrane surface.

## 4. Conclusions

In this paper, a modified electrodialysis (called MASED) with monovalent anion-selective membranes (MVA membranes) and standard cation-exchange membranes was proposed for the fractionation of aqueous mixtures containing monovalent salt (NaCl) and reactive dyes. It was demonstrated that the MASED process allowed the removal of mineral salt from the dye solutions with excellent efficiency (97.4–99.4%) regardless of the dye molecular weight, its concentration, and the amount of NaCl in the experimental solutions. The final diluate solutions (containing retained dyes) were characterized by a low electrical conductivity (around 0.1 mS/cm). On the other hand, a continuous drop in the amount of reactive dye in the diluate cells during MASED was observed, while the movement of dye macro-anions through the MVA membranes to concentrate cells was irrelevant. Dye concentration in the concentrate compartments amounted to 1–2 mg/L. These observations supported the statement that the reactive dyes were vulnerable to adsorption by the MVA membranes during MASED. The amount of dye accumulated in the ED stack depended on the feed dye concentration in the dye–salt mixture—the higher the dye concentration, the more dye particles were adsorbed. This effect was especially pronounced for an elevated salt content in the dye–salt mixtures as well as for the reactive dyes of the highest molecular weight (>900 Da).

It was assumed that dye reactivity was the main parameter influencing the intensity of dye accumulation on/in the MVA membranes. At the end of the MASED process, the loss of dye mass due to its deposition in the ED stack varied from 11 to even 40% (in relation to the initial dye mass in the diluate cells). In turn, the retention of the reactive dyes in the diluate compartments after the MASED treatment varied significantly (from 59 to 81%).

The existence of dye anions on the membrane surface and/or in membrane matrix hindered the migration of monovalent salt anions through the MVA membranes. Consequently, the power demand for salt removal from dye–salt solutions was higher than the power demand for salt removal from solutions containing only salt. Taking into account the mitigation of dye adsorption on the ion-exchange membranes, an integrated, tight ultrafiltration-selective electrodialysis (TUF-MASED) process is recommended for the valorisation of exhausted dye-baths containing reactive dyes.

## Figures and Tables

**Table 1:** Reactive dyes used in MASED.

Dye Name	MW, Da	Reactive Group/Number of Reactive Groups	Dye Symbol	λ_max_ ^1^, nm	Structural Formula
Reactive Orange 16	617.53	Vinyl sulfone/1	RO16	486	C_20_H_17_N_3_Na_2_O_11_S_3_
Remazol Brilliant Blue R	626.53	Vinyl sulfone/1	RBB	594	C_22_H_16_N_2_Na_2_O_11_S_3_
Reactive Orange 20	682.18	Monochloro-triazine/1	RO20	491	C_23_H_16_ClN_7_O_10_S_3_
Reactive Black 5	991.8	Vinyl sulfone/2	RB5	533	C_26_H_21_N_5_Na_4_O_19_S_6_
Reactive Red 120	1469.98	Dichloro-triazine/2	RR120	624	C_44_H_24_Cl_2_N_14_Na_6_O_20_S_6_

^1^ Optimal wavelength for absorbance measurement.

## Data Availability

The original contributions presented in this study are included in the article. Further inquiries can be directed to the corresponding author.
